# Hepatic Infiltration by Splenic Marginal Zone Lymphoma in a Patient With Cured Hepatitis C

**DOI:** 10.7759/cureus.18667

**Published:** 2021-10-11

**Authors:** Leonor Silva, Mafalda Alpoim, Ana Ribeiro, Pedro Caiano Gil, Rute Lopes Caçola

**Affiliations:** 1 Internal Medicine, Centro Hospitalar Vila Nova de Gaia/Espinho, Vila Nova de Gaia, PRT; 2 Hematology Department, Centro Hospitalar Vila Nova de Gaia/Espinho, Vila Nova de Gaia, PRT; 3 Pathology Department, Centro Hospitalar Vila Nova de Gaia/Espinho, Vila Nova de Gaia, PRT

**Keywords:** non-hodgkin lymphoma, marginal zone lymphoma, hepatitis c, liver disease, diffuse large b-cell lymphoma

## Abstract

Splenic marginal zone lymphoma (SMZL) accounts for only 1-2.7% of all lymphomas. Almost all patients have bone marrow (BM) involvement but only one-third has liver involvement. The higher prevalence of hepatitis C virus (HCV) infection in these patients has led to the hypothesis of viral involvement in lymphomagenesis.

In this report, we present a case of a 48-year-old woman, with cured hepatitis C, presenting with fever, weight loss, nausea, abdominal pain, and jaundice. She had leucocytosis with lymphocytosis, a progressively worsening cytocholestasis, and hepatosplenomegaly. Liver biopsy, immunophenotyping, and BM biopsy were performed, resulting in the diagnosis of SMZL. The patient started chemotherapy (rituximab, cyclophosphamide, doxorubicin hydrochloride, vincristine, and prednisolone) with an initial good response, but later progression to high-grade lymphoma and was recommended to undergo salvage chemotherapy followed by auto-transplant.

Despite the unusual liver involvement, we should consider hepatic infiltration by lymphomas, such as SMZL, especially in patients with a history of HCV infection.

## Introduction

Splenic marginal zone lymphoma (SMZL) is an indolent B-cell neoplasia involving the spleen, bone marrow (BM), and blood [1,2]. It represents 1-2.7% of all lymphomas and 10% of non-Hodgkin's lymphomas (NHL) [2-4]. Most patients are asymptomatic, although splenomegaly (75%), adenomegaly, and BM involvement may be present [4]. SMZL has been associated with hepatitis C virus (HCV) infection, raising the hypothesis of viral involvement in lymphomagenesis [5].

We discuss a rare case of a 48-year-old woman, with cured hepatitis C, diagnosed with SMZL presenting with uncommon hepatic infiltration initially and later progression to high-grade lymphoma.

## Case presentation

A 48-year-old Caucasian woman presented with an eight-month history of anorexia, weight loss (25 Kg), nocturnal hypersudoresis, nausea, and food vomiting, which had exacerbated in the last two months. Three days before admission, she had developed intense abdominal pain on both flanks and afternoon-predominance fever (38.5 ºC); she was admitted to the ER.

Her medical history included untreated chronic hepatitis C (diagnosed 16 years ago) and cholecystectomy. She was medicated with alprazolam 1 mg and diazepam 10 mg once daily. She was a current smoker (4.5 pack-years), former drug addict (intravenous heroin usage between 20-28 years of age), and had discontinued her alcohol abuse (one year ago). She had had contact with a vaccinated cat. She denied any recent travel, transfusions, or risky sexual behavior.

In the ER, she appeared slightly jaundiced with abdominal pain on palpation of both flanks with spleen’s inferior pole palpable on the left flank and palpable hepatic edge 3 cm from the costal margin. The remainder of the general examination was normal.

On admission, she had normal haemoglobin (Hb) (12.6 g/dL) and platelet count (156 x 10E3/uL) with leucocytosis (13.9 x 10^3^/uL). Lactate dehydrogenase (LDH) (690 U/L), total bilirubin (TB) (1.72 mg/dL), direct bilirubin (DB) (1.30 mg/dL), glutamic-oxalacetic transaminase (GOT) (196 U/L), glutamic-pyruvic transaminase (GPT) (224 U/L), and C-reactive protein (CRP) (6.80 mg/dL) were elevated. Coagulation study [international normalized ratio (INR): 1.16, activated partial thromboplastin time (aPTT): 31 seconds], ionogram, and renal function were normal.

The patient was admitted to the Internal Medicine Service for further investigation. During hospitalization, she maintained fever, nausea, vomiting, and abdominal pain, with a progressive exacerbation of cholestasis (maximum TB: 12.7 mg/dL, DB: 12.0 mg/dL, alkaline phosphatase: 826 U/L, and gamma-glutamyl transferase: 157 U/L), although GOT/GPT returned to normal levels. Leukocytosis reached 15.3 x 10^3^/uL with lymphocytosis. CRP increased to a maximum of 25 mg/dL and procalcitonin was 0.78 ng/mL (Table 1). She had hypoalbuminemia.

**Table 1:** Blood exams including admission and maximum values during inpatient investigation

Variables	Admission results	Maximum values	Normal values
Leukocytes	13.9 x 10^3^/uL	15.3 x 10^3^/uL	3.6–11 x 10^3^/uL
Lymphocytes	14.7 x 10^3^/uL	14.9 x 10^3^/uL	1–4.8 x 10^3^/uL
Lactate dehydrogenase (LDH)	690 U/L	851 U/L	135–214 U/L
Total bilirubin (TB)	1.72 mg/dL	12.7 mg/dL	0.1–1.1 mg/dL
Direct bilirubin (DB)	1.30 mg/dL	12 mg/dL	0.1–0.3 mg/dL
Glutamic-oxalacetic transaminase (GOT)	196 U/L	224 U/L	4–27 U/L
Glutamic-pyruvic transaminase (GPT)	224 U/L	285 U/L	4–34 U/L
Alkaline phosphatase	469 U/L	826 U/L	35–104 U/L
Gamma-glutamyl transferase	162 U/L	157 U/L	5–61 U/L
Albumin	1.9 g/dL	2.9 g/dL	3.4–4.8 g/dL
Procalcitonin	---	0.78 ng/mL	<0.5 ng/mL
C-reactive protein (CRP)	6.80 mg/dL	25 mg/dL	<0.5 mg/dL

Serum protein electrophoresis and immunoglobulin assay were normal and there was no complement consumption. Antinuclear antibodies presented a homogeneous pattern (titer: 1/160). Antineutrophil cytoplasmic, anti-hepatic, anti-mitochondrial, anti-double-stranded DNA, and anti-extractable nuclear antigens antibodies were negative.

Serologies showed a positive HCV antibody with undetectable viral load, previous hepatitis A and cytomegalovirus infection, and reactive varicella-zoster IgM antibody (non-reactive IgG antibody). HIV, hepatitis B virus (HBV), Epstein-Barr virus, Treponema pallidum, Leptospira, Borrelia, and mononucleosis serological tests were negative. Blood and urine cultures were also negative.

**Figure 1:**
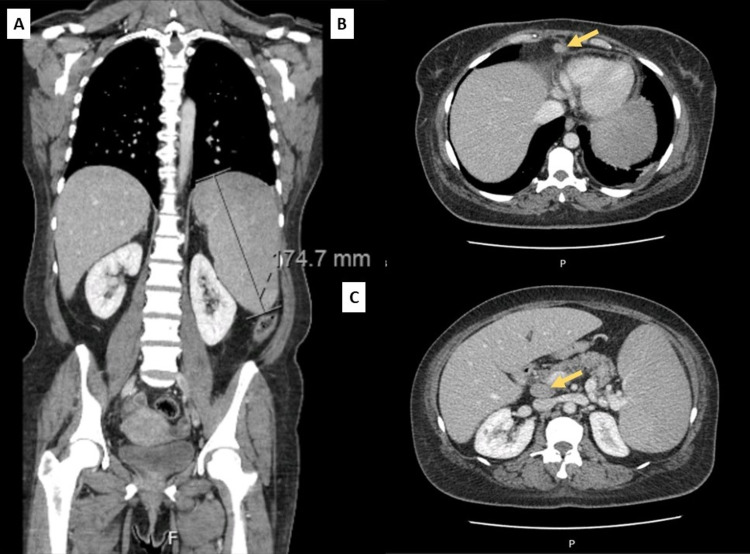
Cervical-thoracic-abdominal-pelvic CT scan A. Suggestive features of chronic liver disease, splenomegaly (17.5 cm), and signs of portal hypertension; discreetly prominent main bile duct (10 mm) secondary to cholecystectomy. B. Ganglion formation (18 x 11 mm) anteriorly to the pericardium. C. Several ganglia in the hepatic hilum (largest: 28 x 17 mm) CT: computed tomography

**Figure 2:**
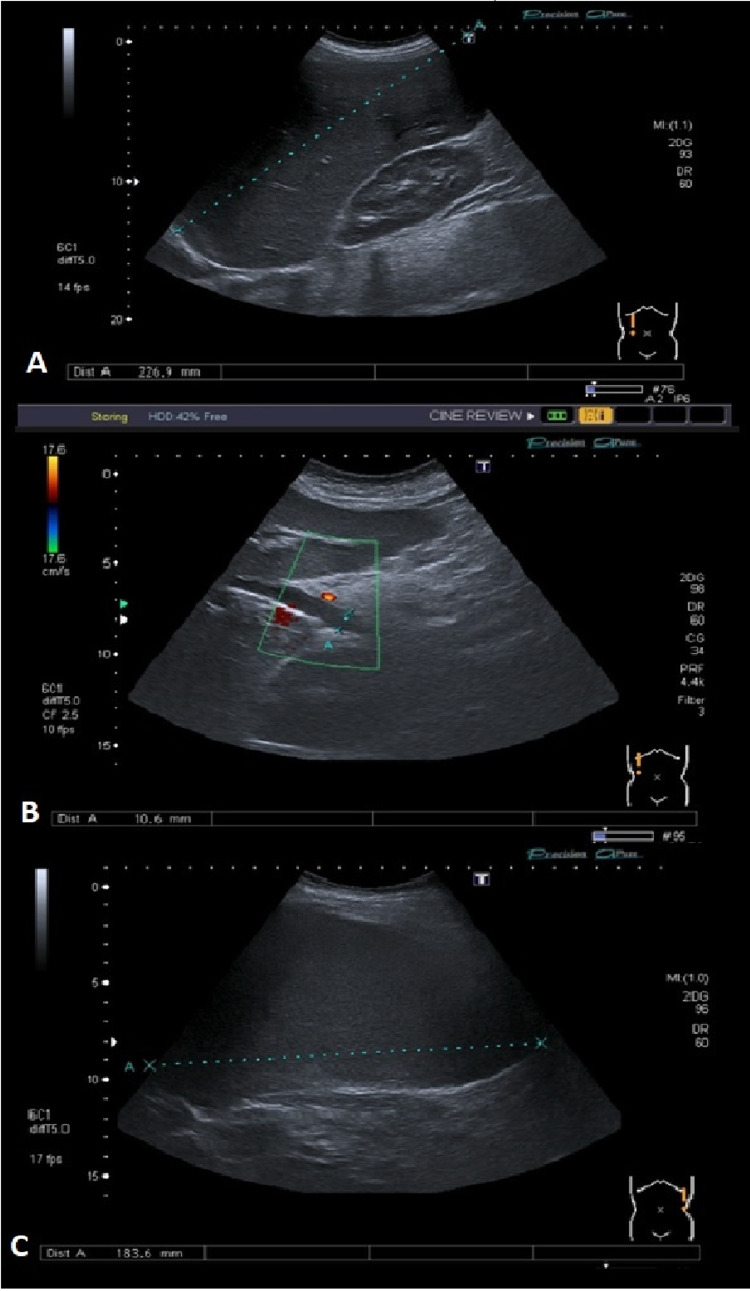
Abdominal ultrasound at ER admission A. Hepatomegaly (22.7 cm). B. Slight prominence of the intrahepatic biliary tract and main biliary ectasia (10 mm) without obstructive cause. C. Globular splenomegaly (18 cm) ER: emergency room

Cervical-thoracic-abdominal-pelvic CT scan (Figure 1) confirmed the pathologic findings in abdominal ultrasound (Figure 2), revealing a ganglion formation (18 x 11 mm) anteriorly to the pericardium, suggestive features of chronic liver disease, splenomegaly (17.5 cm), and signs of portal hypertension, as well as discreetly prominent main bile duct (10 mm) secondary to cholecystectomy and several ganglia in the hepatic hilum (largest: 28 x 17 mm).

**Figure 3:**
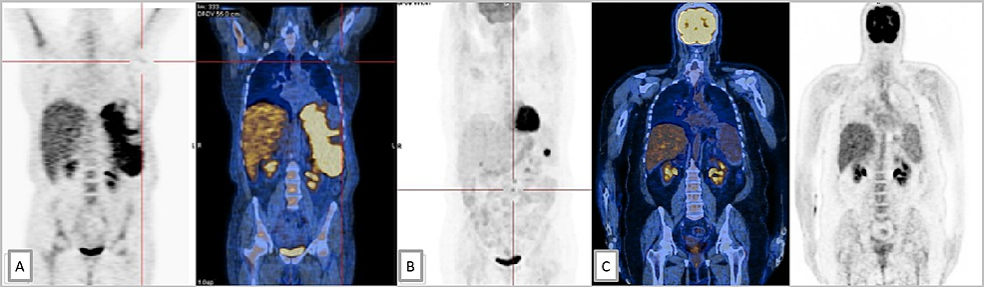
PET scan at diagnosis and revaluation PET scans A. PET scan at diagnosis. B. Revaluation PET scan after six cycles of R-CHOP. C. PET scan two months after chimeric antigen receptor T-cell therapy showing complete remission PET: positron emission tomography; R-CHOP: rituximab, cyclophosphamide, doxorubicin hydrochloride, vincristine, and prednisolone

PET scan (Figure 3A) presented intense anomalous 18F-fluorodeoxyglucose (FDG) uptake, with heterogeneous distribution in the spleen [maximum standardized uptake value (SUV): 7.7] and discrete increased uptake in humerus, pelvis, and femur diaphyses. There was no FDG uptake in the liver.

Peripheral blood (PB) smear (Table 2) showed lymphocytes with cytoplasmic extensions and irregularly nucleated mononuclear cells, chromatin laxa, and rare vacuolization. PB immunophenotyping demonstrated a monoclonal small B-lymphoid population (99%) with SMZL-compatible immunophenotyping.

**Figure 4:**
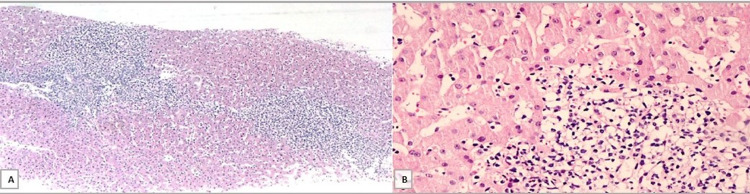
Hepatic biopsy - hematoxylin and eosin (H&E) A. Hepatic parenchyma with nodular lymphoplasmacytic infiltrate centered in the portal spaces ('hepatitis-like' and extension to the adjacent parenchyma) (10x). B. The lymphoplasmacytic population has predominantly small cells (40x)

**Figure 5:**
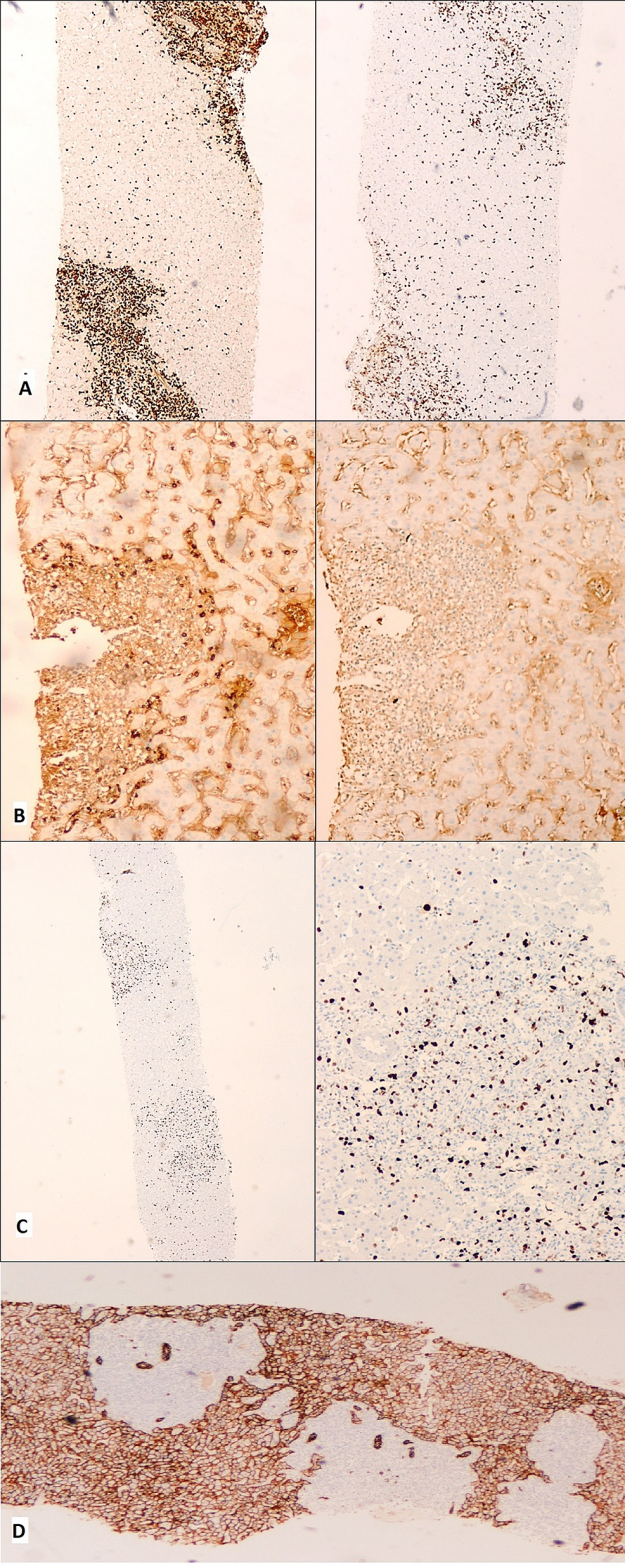
Liver biopsy (immunochemistry) A. Left (4x): PAX-5; right (4x): CD3; there is a predominance of B lymphocytes. B. Left (10x): light chain lambda; right (10x): light chain kappa; the lambda predominance is striking. C. Ki67/MIB-1 immunostain (left 2x, right 20x); the proliferation index is low. D. Aberrant CD138 expression in epithelium evidences a ‘hepatitis-like’ pattern of the infiltrate (4x)

Hepatic biopsy (Table 2; Figures 4, 5) presented a preserved trabecular architecture, slight fibrosis of portal spaces with portal space expansion by atypical lymphoid population, ductulitis lesions, and mild/moderate interface lesions. It also showed a small lymphocyte population with PAX-5 and BCL2 expression and a small plasmacytic population with CD138, MUM-1, and lambda light chain expression restriction. A low proliferation index was observed based on Ki67/MIB-1 immunostain (Figure 5C). The immunophenotyping revealed a monoclonal lymphoid B population with lambda light chain and strong CD11c expression.

**Figure 6:**
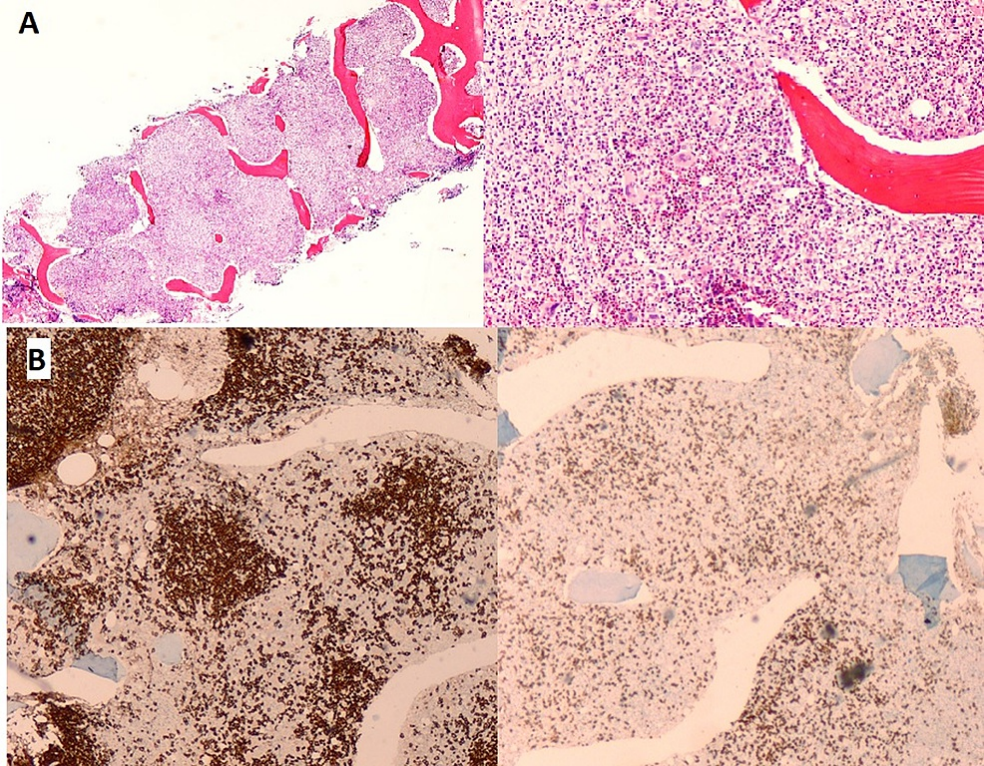
Bone marrow trephine biopsy A. (H&E) on the left side (2x), there is hypercellular medullary space. On the right side (20x), hematopoietic tissue is present with architectural disarray. B. (immunohistochemistry, 20x) CD20 (left) and CD3 (right); it is evident that a dense lymphoid infiltrate, predominantly B-cell, with nodular and paratrabecular distribution is present H&E: hematoxylin and eosin

BM biopsy (Table 2; Figure 6) showed hypercellularity with atypical nodular small lymphocytes infiltration (60%) expressing CD20 and PAX-5, compatible with infiltration by SMZL.

**Table 2:** Histological, immunochemistry, and immunophenotyping results of peripheral blood smear and liver, subcutaneous nodule, lymph node, and bone marrow biopsies

	Histology	Immunochemistry	Immunophenotyping
Peripheral blood smear	Lymphocytes with cytoplasmic extensions; irregularly nucleated mononuclear cells with chromatin laxa and rare vacuolization; Gumprecht patches		Monoclonal small B-lymphocytes (99%): immunophenotyping compatible with marginal zone splenic non-Hodgkin's lymphoma B
Hepatic biopsy	Slight fibrosis; portal spaces: 1) expansion by atypical lymphoid population; 2) ductulitis lesions; 3) mild to moderate interface lesions	Atypical lymphocyte population: PAX-5 and BCL2 expression; no expression of CD10, CD5, BCL6, CD23, or cyclin D1. Small plasmacytic population: CD138 and MUM-1 expression; restriction of lambda light chain expression	Monoclonal lymphoid population (52%): strong CD11c expression; lambda light chain expression; no expression of CD5 or CD10
Bone marrow biopsy	Hypercellularity; atypical nodular lymphoid infiltration; predominance of small lymphocytes	Small lymphocytes (60%): CD20 and PAX-5 expression; no expression of CD3, CD5, CD10, BCL6, BCL2, CD23, cyclin D1, or annexin A	
Subcutaneous nodule biopsy			B lymphocytes population (56%): CD19, CD20, CD38, CD81, BCL2, and lambda light chain expression; no expression of CD5 or CD10
Lymph node biopsy	Large and atypical lymphoid cells tissue infiltration; several areas of necrosis	Large, atypical lymphoid population: PAX-5, CD79A, CD38, and MUM-1 expression; CD23 expression does not show dendritic follicular network; no expression of CD20, CD3, BCL2, BCL6, CD10, c-MYC, CD5, CD3, or cyclin D1	

B symptoms and splenomegaly favored lymphoma diagnosis. Jaundice and cytocholestasis are uncommon; however, portal space expansion conditioning ductilitis and interface lesions may justify these findings. Hepatic, BM, and PB immunophenotyping demonstrated the presence of monoclonal B-lymphoid population expressing CD20, PAX-5, BCL2, and CD11c, all frequent in SMZL and lymphoplasmacytic lymphoma. CD138/MUM-1 small plasmacytic population with lambda light chain expression restriction may be present in both lymphomas. The absence of CD10 and BCL6 excluded follicular lymphoma and the absence of CD5 and cyclin D1 excluded mantle cell lymphoma [1,6]. Hairy cell leukemia was ruled out as CD103 and CD25 were negative [7]. Since serum protein electrophoresis and immunoglobulin were normal, associated with small lymphocytes with cytoplasmic extensions and atypical nodular lymphoid infiltration in BM, SMZL seemed the most likely diagnosis.

In the presence of a rapid clinical and analytical deterioration, the patient was started on prednisolone (60 mg/day for eight days) resulting in nausea and abdominal pain resolution with a slight improvement in cytocholestasis, despite maintaining fever. Due to the suspicion of transformation to diffuse large B-cell lymphoma (rapid clinical progression and high LDH and SUV), combined with splenic biopsy risks, she was also started on R-CVP (rituximab, cyclophosphamide, vincristine sulfate, prednisone). After the first cycle, she developed anaemia (Hb: 6.3 g/dL), leukopenia (2.21 x 10^3^/uL), and thrombocytopenia (12 x 10^3^/uL). She was discharged from inpatient service, continuing treatment in a day hospital unit. Twenty-one days after R-CVP initiation, the patient underwent the first cycle of R-CHOP (rituximab, cyclophosphamide, doxorubicin hydrochloride, vincristine, prednisolone) with a 50% reduction in doxorubicin and vincristine. The second cycle of R-CHOP had 100% of the doxorubicin dose and 50% of the vincristine dose, while the third and fourth cycles were full doses. The fifth and sixth cycle, due to neuropathy, was performed with vincristine 1 g. The patient initially had a good response, being asymptomatic, without fever or pancytopenia with improved cytocholestasis [normal GOT, GPT, and bilirubin; high AP (117-173 U/L)] and normalization of inflammatory markers six months after discharge. HCV viral load remained negative three months after discharge.

Despite the initial good response, the disease progressed to high-grade lymphoma. Revaluation PET (Figure 3B) showed a reduction of the affected areas but intensely avid FDG foci in the spleen (qSUVmax=12.2) and lateroaortic adenopathies, reflecting refractory disease.

**Figure 7:**
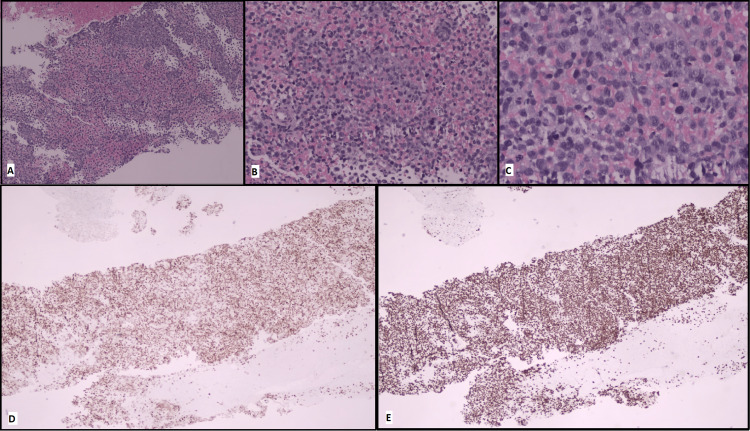
Cervical lymph node biopsy A. Lymph node completely replaced by atypical lymphoid infiltration (H&E, 4x); no normal lymph node is observed. B. Atypical cells are medium to large with abundant images of apoptosis (H&E, 10x). C. Atypical cells are medium to large with centroblastic morphology (H&E, 20x). D. Expression of PAX-5 is noticed in most cells (immunohistochemistry, 4x). No expression of CD20 is observed (secondary to administration of rituximab?). E. Cells presents an expression of MUM-1 in the absence of CD10 and BCL6 expression (non-GCB immunophenotype – Hans algorithm) (immunohistochemistry, 4x) H&E: hematoxylin and eosin

Three months after the sixth R-CHOP cycle, the patient presented with stiff and painful subcutaneous nodules at occipital, abdominal, and infra-mammary regions. Subcutaneous nodule biopsy immunophenotyping (Table 2) and cervical lymph node biopsy were performed, revealing a diffuse large B-cell lymphoma (Figure 7).

In the presence of a high-grade lymphoma under first-line treatment, the patient accomplished two cycles with ifosfamide, etoposide, and carboplatin with persistent disease, and then underwent rituximab, dexamethasone, cytarabine, and cisplatin followed by chimeric antigen receptor T-cell therapy. Two months later, the PET showed complete remission (Figure 4C).

## Discussion

SMZL is a rare B-cell neoplasia with a female predominance, and the average age at diagnosis is between 65-69 years [2-4,6]. The median overall survival is 10-11 years [4]. BM involvement is common at diagnosis but only one-third has liver involvement [3]. Hepatomegaly is less common and lymphadenopathy is rare (abdominal: 25%) [3,7]. Anaemia, thrombocytopenia, and leucocytosis occur in 25% of patients [3].

The prevalence of HCV in patients with SMZL is higher than that in the general population, and patients with HCV presented with SMZL in 4.2% of cases [4,8]. Although the mechanism is not completely understood, lymphoma development may be related to HCV chronic antigenic stimulation [9]. The causal role of HCV in lymphomagenesis is supported by the phenomenon of lymphoma regression after HCV eradication [2].

Our patient, at diagnosis, had spontaneously cured hepatitis C. Although the exact date of cure is unknown, we speculate whether the risk of developing SMZL in such patients is similar to the general population or higher due to previous HCV infection. On the other hand, given that the disease is cured in some cases after hepatitis C treatment, we also wonder whether the mechanism behind the cure may be related to the pharmacological mechanisms as well as HCV eradication.

Differential diagnosis with respect to lymphoplasmacytic lymphoma is difficult as SMZL may present with plasmacytic differentiation and serum monoclonal paraproteinemia (28%) [1,3]. If lymphocytes are organized in marginal zone pattern or intrasinusoidal involvement, as observed in our patient's BM biopsy, SMZL should be suspected [3].

SMZL does not have a specific immunophenotyping (usually positive for CD20, CD79a, PAX-5/BSAP, IGM, and BCL2 surface immunoglobulins and negative for CD5, CD10, BCL6, cyclin D1/BCL1, CD43, annexin A1, LEF1, CD103, and CD123); therefore, it is often a diagnosis of exclusion [1,2].

There is no consensus on the treatment of this condition: splenectomy, chemotherapy, rituximab, or antiretroviral treatment may be employed in HCV patients [2]. Due to the significant morbidity associated, risk of infection, and no impact on BM disease, splenectomy may be an unreasonable option [10-13]. Rituximab monotherapy has been associated with high response rates, higher five-year progression-free survival rates, and a favorable safety profile [10-15].

## Conclusions

Although liver involvement in SMZL is uncommon, this diagnosis should be considered in patients with hepatic abnormalities, especially if associated with HCV infection. SMZL is usually indolent; however, sometimes, there is transformation into high-grade lymphomas, which are more aggressive and associated with shorter survival periods.
